# Hsp70 and Hsp110 Chaperones Promote Early Steps of Proteasome Assembly

**DOI:** 10.3390/biom13010011

**Published:** 2022-12-21

**Authors:** Ana C. Matias, Joao Matos, R. Jürgen Dohmen, Paula C. Ramos

**Affiliations:** 1 Center of Molecular Biosciences, Institute for Genetics, Department of Biology, Faculty of Natural Sciences and Mathematics, University of Cologne, 50674 Cologne, Germany; 2 Departamento de Química e Bioquímica, Faculdade de Ciências e Tecnologia, Universidade do Algarve, 8000-117 Faro, Portugal

**Keywords:** proteasome biogenesis, chaperones, Hsp70, Hsp110, Ssa1, Sse1

## Abstract

Whereas assembly of the 20S proteasome core particle (CP) in prokaryotes apparently occurs spontaneously, the efficiency of this process in eukaryotes relies on the dedicated assembly chaperones Ump1, Pba1-Pba2, and Pba3-Pba4. For mammals, it was reported that CP assembly initiates with formation of a complete α-ring that functions as a template for β subunit incorporation. By contrast, we were not able to detect a ring composed only of a complete set of α subunits in *S. cerevisiae.* Instead, we found that the CP subunits α1, α2, and α4 each form independent small complexes. Purification of such complexes containing α4 revealed the presence of chaperones of the Hsp70/Ssa and Hsp110/Sse families. Consistently, certain small complexes containing α1, α2, and α4 were not formed in strains lacking these chaperones. Deletion of the *SSE1* gene in combination with deletions of *PRE9* (α3), *PBA3*, or *UMP1* genes resulted in severe synthetic growth defects, high levels of ubiquitin-conjugates, and an accumulation of distinct small complexes with α subunits. Our study shows that Hsp70 and Hsp110 chaperones cooperate to promote the folding of individual α subunits and/or their assembly with other CP subunits, Ump1, and Pba1-Pba4 in subsequent steps.

## 1. Introduction

The ubiquitin/proteasome system (UPS) of eukaryotic cells provides an essential proteolytic control system, malfunctions of which are linked to various human diseases [1,2]. A central component of the UPS is the 26S proteasome, a multimeric protease complex composed of the 20S proteasome, also called catalytic core particle (CP), and of 19S regulatory particles (RP) [3]. Numerous observations have linked malfunctions of the proteasome or an impairment of its biogenesis to various disease states including cancer, neurodegeneration, autoinflammatory syndromes, or cardiomyopathies, as well as to aging [4,5,6,7]. The assembly of CPs and RPs is independently promoted by various dedicated and specific assembly chaperones [8,9,10,11]. The mature and active CP is structurally organized as four stacked heptameric rings, two α-rings flanking two β-rings, the latter of which form the inner chamber bearing the six catalytic sites [12]. Eukaryotic CPs assemble from 14 distinct subunits. The efficiency of this process depends on the dedicated chaperones Ump1, Pba1-Pba2, and Pba3-Pba4 in yeast (called UMP1/POMP, PAC1-PAC2, and PAC3-PAC4 in mammals). Vertebrates can assemble variants of the proteasome (immunoproteasome and thymoproteasome) with slightly different cleavage specificities by incorporating paralogues β subunits [10]. In addition, the formation of alternative proteasomes can occur when a second copy of α4 is incorporated in place of α3 during CP assembly [13,14], or when an alternative α4 subunit is incorporated (spermatoproteasome) [15]. Most prokaryotic CPs, in contrast, assemble from only two or four distinct subunits and, in general, without the assistance of specific chaperones [9,10,11,15]. A well-characterized intermediate in CP assembly is a half-CP precursor complex, also called 15S PC, in yeast containing the chaperones Ump1 and Pba1-Pba2, as well as 13 out of the 14 subunits [16,17,18,19,20]. CP formation by dimerization of two such complexes is triggered by incorporation of β7 subunits, the C-terminal extensions of which reach out to the respective other halves to stabilize the newly formed 20S particle [17,21]. Recently, we reported that β7 recruitment is promoted by an N-terminal domain of Ump1 that interacts with the β7 pro-peptide [22]. Upon maturation of the active sites by autocatalytic processing, the assembly chaperones Ump1 is degraded, and Pba1-Pba2 is released [9,19]. Although 15S PC formation, as well as its dimerization into 20S RP, are steps common to the CP assembly pathways in all species from archaea to mammalians, the details of the pathways leading from single subunits to the 15S PC seem to vary substantially between the species. Early studies with the archaeon *Thermoplasma*, the proteasome of which is formed only by one kind of α and β subunits, led to the proposal of a biogenesis model according to which the formation of a homo-heptameric ring of α subunits would create a platform for β ring incorporation [23]. However, in the eubacteria *Rhodococcus*, with a more complex proteasome composed of two distinct α and two different β subunits, this model apparently does not apply. Instead, α-β dimers were initial intermediates leading to 15S PC formation [24]. Another report showed that, although the α subunits from the archaeon *Methanococcus maripaludis* are able to form single ring structures, functional proteasomes were even assembled from mutant α subunits incapable of forming such α rings indicating that assembly occurred in a α ring-independent manner [25]. In mammals, several studies suggested that the assembly of rings composed of seven distinct α subunits is promoted by two heterodimeric chaperones: PAC1-PAC2 (homolog of the yeast Pba1-Pba2) and PAC3-PAC4 (homolog of yeast Pba3-Pba4). These rings are thought to serve as a template for subsequent β subunit assembly [26,27]. In the model organism *S. cerevisiae*, the proteasome of which is also constituted of 14 distinct subunits, there is no report identifying α-rings as assembly intermediates. Instead, precursors containing Ump1 [22] and β2, but not Pba1-Pba2, were detected [19]. More recently, the isolation of a subcomplex containing a subgroup of α and β subunits has been reported [28].

In the present work, we dissected early assembly steps that lead to the 15S PC in yeast. We found that α1, α2, and α4 subunits assemble into small complexes, formation of which depends on Hsp70 and Hsp110 chaperones. These complexes accumulate either in the absence of the α3 subunit, or when the dedicated chaperones Ump1 or Pba1-Pba4 are missing. The Hsp70-Hsp110 chaperone system likely promotes the correct folding of individual subunits and/or the assembly with other subunits and dedicated chaperones.

## 2. Materials and Methods

### 2.1. Yeast Strains and Media

Yeast strains used in this work are listed in Table S1. Yeast rich (YPD) and synthetic (SD) minimal media with 2% dextrose were prepared as described previously [29]. Strains expressing *PRE6* and *SCL1* under the control of the P*_GAL_* promoters were grown to an OD_600_ of 0.6 in media containing galactose, washed with sterile distilled water, resuspended in media containing glucose, and further incubated for the indicated periods of time. Spot assays were prepared as follows: 2 mL of overnight cultures were diluted in 5 mL of the appropriate media and grown until an optical density at 600 mm (OD_600_) of about 0.8 was reached. The cultures were then diluted with sterile water to OD_600_ of 0.5 in a total volume of 200 μL. From this suspension, sequential 5 μL of 1:10 dilutions were spotted onto plates, and the cells allowed to grow for 1–3 days. An *SSE1* disruption cassette was obtained by amplifying the *TRP1* gene from pFA6a-TRP1 [30]. The *sse1∆::TRP1* strain (PR93) was prepared by gene replacement in the strain JD47-13C. Construction of a set of strains, each with an individual endogenous chromosomal locus of one of the 14 CP subunits encoding genes modified such that a C-terminally tagged version is expressed, was performed as follows. Using primers that contained flanking EcoRI and KpnI sites, 5’∆ fragments of the respective genes were amplified by PCR. These sites were then used to insert the amplified fragments into integrative plasmids containing sequences encoding epitope tags followed by the terminator sequence of the *CYC1* gene (T*_CYC1_*). These plasmids were based upon YIplac128 (*LEU2* marked), YIplac211 (*URA3* marked), or YIplac204 (*TRP1* marked) [31]. Each of the resulting plasmids was linearized with a restriction enzyme that cleaved within the coding sequence of the CP gene for targeted integration into the *S. cerevisiae* genome. The resulting strains contained one copy of the respective gene expressed from its natural promoter and fused in-frame to the tag-coding sequence, followed by T*_CYC1_*, in addition to a 5’∆ fragment of the same gene without promoter. The epitope tags used were 2xHA (“HA”) or FLAG-6His (“FH”) [29]. All plasmids were verified by DNA sequencing. Shutdown strains were prepared by exchanging native promoters of *PRE6* and *SCL1* with the P*_GAL1_* promoter (P*_GALS_*) [32]. In these cases, a fragment from pYM-N30 plasmid [33] containing 45 bp of the CP gene’s promoter sequence (5′ flanking region) and 45 bp of the 5′ end of the CP gene’s ORF, including the START codon (3′ flanking region), was amplified by PCR. The resulting PCR product was used to transform yeast cells selecting for G418-resistant clones (*kanMX4* marker). Correct insertion of P*_GALS_* in front of the desired genes was verified by analytical PCR [34]. 

### 2.2. Protein Extraction 

Total protein crude extracts analyzed by native electrophoresis or by gel filtration were prepared in 26S buffer (50 mM Tris-HCl, pH 7.5, containing 1 mM DTT, 5 mM MgCl_2_, 2 mM ATP, and 15% glycerol) [16]. For a native PAGE analysis, yeast cells from 15 mL exponentially growing cultures (OD_600_ 0.8–1.2) were harvested at 3000× *g* rcf, washed with cold water, frozen in liquid nitrogen, and stored at −80 °C. The cells were lysed with glass beads (0.4–0.5 mm, Sigma-Aldrich Chemie GmbH, Taufkirchen, Germany) in extraction buffer by vortexing. Cell debris was removed by centrifugation at 15,800× *g* rcf at 4 °C. The protein content in the supernatant was determined using the Bradford protein assay from Bio-Rad (Hercules, CA, USA). For gel filtration studies, yeast cells from 200 mL exponentially growing cultures were collected by centrifugation (OD_600_ 0.8–1.2). Extraction buffer was added in a proportion of 2 mL/g of pelleted yeast cells. Cell paste was ground to powder in a mortar in the presence of liquid nitrogen. After centrifugation at 31,000× *g* rcf for 10 min at 2 °C, the supernatant was subjected to a second centrifugation at 60,000× *g* rcf for 30 min at 4 °C. The protein concentrations in the extracts in parallel experiments were adjusted to 5 mg/mL using extraction buffer.

### 2.3. Fractionation of Whole-Cell Extracts by Gel Filtration

Samples containing 1 mg of total protein in a total volume of 200 μL were separated on a Superdex200 column equilibrated with 26S buffer and coupled to an ÅKTA FPLC (GE healthcare Germany, Solingen). The flow rate was 0.35 mL/min, and fractions of 0.5 mL were collected. The Superdex200 column was calibrated using the following standards: ferritin (440 kDa), catalase (232 kDa), bovine serum albumin (67 kDa), and ovalbumin (43 kDa); dextran blue was used to monitor the void volume (GE healthcare).

### 2.4. Immunoaffinity Purification of the α4 Complex and Mass Spectrometry Analysis

Cells from the strain JM12 expressing α4/Pre6-FH were grown at 30 °C in a culture volume of 4 L in SD without tryptophan until an OD_600_ of 1.2. The cells were pelleted and frozen in liquid nitrogen. The cells were reduced to powder with the help of a mortar in the presence of liquid nitrogen with 26S buffer with protease inhibitors (Complete, EDTA-free, Roche Diagnostics, Mannheim, Germany) in the proportion 2 mL/g wet weight. Cell debris were removed by centrifugation at 30,000× *g* rcf for 30 min at 4 °C. The supernatant was subjected to a buffer exchange with the help of a PD-10 column (GE healthcare) equilibrated with FLAG buffer (50 mM Tris-HCl, 150 mM NaCl, pH 7.4). The eluted proteins were added to 1 mL of anti-FLAG resin equilibrated with 15 mL FLAG buffer for 2 h and 30 min in batch with rotation at 4 °C. The suspension was transferred to an empty column. The flow-through was collected, and the FLAG resin was washed 3 times with 20 mL FLAG buffer. The bound proteins were eluted sequentially with 1 mL elution buffer containing FLAG peptide at a concentration of 50 µg/mL, 100 µg/mL, and 200 µg/mL. The eluted material was concentrated with a 15 mL Centricon Biomax 5K (Merck, Darmstadt, Germany). Two hundred microliters of the concentrated protein solution were injected in a Superdex200 column equilibrated with 26S buffer. Proteins from the relevant steps were analyzed by SDS-PAGE. After silver staining, the detected bands were excised and analyzed by LC-MS at the Centre of Molecular Medicine Cologne (CMMC) Central Bioanalytics Unit (ZBA).

### 2.5. Electrophoresis and Immunoblotting

Native samples, freshly prepared, were diluted in native gel sample buffer (240 mM Tris-HCl, pH 8.8, containing 80% glycerol and 0.04% (*m*/*v*) bromophenol blue). Eight micrograms of total protein per lane were applied in Tris-HCl 4–15% gradient gels (Ready Gel, Bio-Rad). The native PAGE was run in an ice box at 16 mA, until the bromophenol blue dye reached the edge of the glass plate in 25 mM Tris-HCl, 192 mM glycine, pH 8.3. The gels were incubated for 15 min in transfer buffer (25 mM Tris, 192 mM glycine, containing 0.1% (*m*/*v*) SDS, and 20% (*v*/*v*) methanol) containing additionally 1% (*m*/*v*) SDS. The proteins were blotted at 0.8 mA/cm^2^ for 2 h. For SDS-PAGE, the protein samples were boiled for 3 min in the presence of 2% SDS and 0.1 M 2-mercaptoethanol, then subjected to 12% SDS-PAGE, and thereafter transferred onto a PVDF membrane (Millipore) in a dry blot system (GE healthcare). The blots were incubated with either mouse anti-ubiquitin P4D1 (Santa Cruz Biotechnology, Dallas, TX, USA), mouse anti-HA 16B12, rat 3F10-POD conjugated, or M2 anti-Flag monoclonal antibodies (all from Merck, Darmstadt, Germany) and were processed as described [16]. 

## 3. Results

### 3.1. Identification of Small Intermediates Containing Either α1, α2, or α4 Subunits

**Figure 1:**
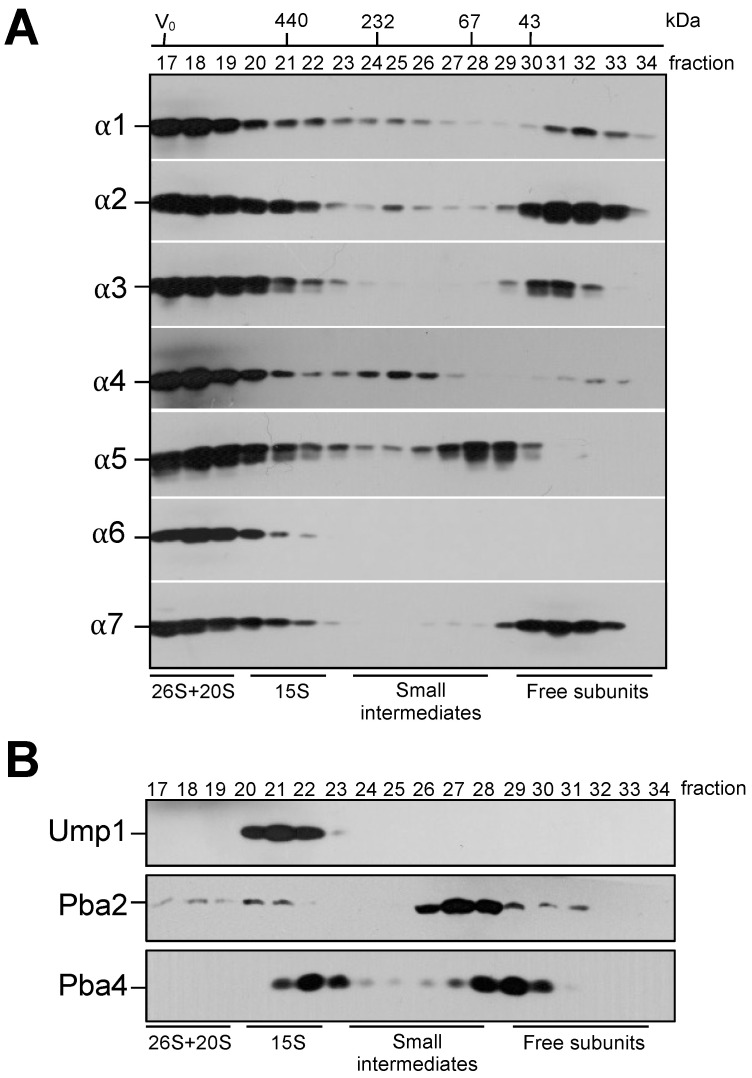
Separation of proteasomal complexes and intermediates according to their molecular weight. To define the distribution by size of the indicated tagged polypeptides in proteasome intermediates and complexes, crude extracts from strains expressing 2xHA tagged versions of the indicated α subunits (**A**) or proteasome-dedicated chaperones (**B**) were separated in a Superdex200 gel filtration column. Fractions were subjected to SDS-PAGE and anti-HA Western blotting. The numbers of fractions (17 to 34) used in the analysis, and the positions of molecular weight standards (kDa) used to calibrate the column are indicated at the top of the panel. Peak positions of 26S and 20S proteasomes, 15S PCs, small intermediates and free subunits are indicated at the bottom.

Using *S. cerevisiae* as a model system, we wanted to dissect the initial steps of proteasome biogenesis. Specifically, we focused on the assembly of individual subunits leading to the 15S precursor complex (PC). Smaller assembly intermediates en route to the 15S PC can be well analyzed in a gel filtration column such as Superdex200 that separates globular proteins in the range of 10–600 kDa. We prepared distinct strains each containing a C-terminally HA-tagged version of one of the 14 CP subunit. From each strain, we analyzed crude extracts by gel filtration. Complexes such as 20S CP or 26S proteasomes were eluted largely in the void volume of the column (Figure 1A, Figures S1 and S2), whereas the 15S PC eluted in fractions 20–22. In parallel, we performed the analysis of tagged dedicated proteasome chaperones in the same conditions (Figure 1B). We divided our gel filtration profile analysis in three molecular weight ranges: (a) complexes larger than 400 kDa (fractions 17–22) comprising 15S PC, 20S CP, and the 26S proteasome; (b) free subunits, smaller than 50 kDa (fractions 29–34); and (c) complexes that correspond to intermediates smaller than 15S PC with molecular weights (MW) of 70–400 kDa (fractions 23–28). As expected, all α subunits were present in complexes with molecular weights higher than 400 kDa, confirming their presence in complexes such as 15S PC, 20S CP, and 26S proteasomes (Figure 1A). Subunits α1, α2, α3, and α7, however, were also detectable in relatively large amounts in fractions comprising proteins with MWs lower than 50 kDa, probably reflecting soluble free subunits. The α5 subunit, instead, was present in fractions with proteins of an MW around 70 kDa, a bit larger than the free form. In this complex, the α5 subunit is likely associated with the chaperone Pba3-Pba4 [35] or with Pba1-Pba2 that peak in the same fractions (Figure 1B). Only small amounts of subunit α4 were detected in the free form. Instead, larger quantities were found in a complex with MW below 230 kDa. Curiously, subunit α6 was not found in any fraction corresponding to proteins with MWs smaller than 400 kDa. To confirm this observation, we HA-tagged α4 and α6 in the same strain and observed that, indeed, the α6 subunit has a profile distinct from α4 (Figure S1). A possible explanation is that α6 is expressed in lower amount than the other α subunits and so could be a rate-limiting subunit in the biogenesis process. In the molecular weight range from 70 to 400 kDa, we detected complexes comprising the subunits α1, α2, α4, and α5, and in lower amounts, α7. In contrast, clear signals were not detected in this molecular weight range for α3 and α6. Taken together, in our assay conditions, we were not able to detect a ring containing all the α subunits in wild-type yeast cells; nevertheless, we found prominent smaller complexes containing α1, α2, and α4. 

When the profiles of the β subunits were analyzed in the same conditions, they were either present in free form, or in the larger assemblies 15S PC, 20S CP, and 26S proteasomes (Figure S2). Here, it should be noted that C-terminal 2xHA tagging of β3 and β6 strongly impaired cell growth, whereas all other strains grew like the wild type (WT). Together, the findings from these analyses indicated that, in contrast to β subunits, some α subunits are present in lower molecular weight intermediates. The dedicated proteasome chaperone Ump1 is confined to the 15S PC fractions (20–22). Pba2, in contrast, was not only found in fractions corresponding to the 15S PC but additionally detected in earlier fractions (corresponding to 20S CP) and in complexes with lower MWs (fractions 26–29). The profile of Pba4 curiously showed one peak detected in fractions 22–23 (around 350 kDa) and a second peak in fractions 28–30 (Figure 1B). The observed distinct elution patterns of Pba2 and Pba4 do not fit the idea that Pba1-Pba2 and Pba3-Pba4 chaperones would simultaneously be associated with an α-ring. By contrast, both Pba chaperones co-fractionated with α5 in fraction 28 (Figure 1).

### 3.2. Isolation and Characterization of Complexes Containing Subunit α4

**Figure 2:**
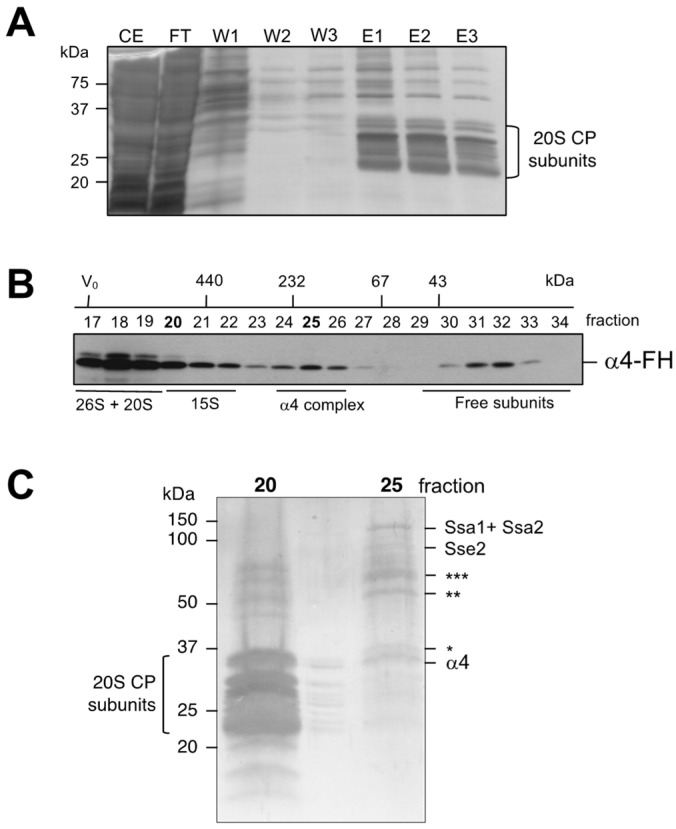
Purification of a low MW complex containing α4 subunit. (**A**) A strain expressing α4-FH was utilized to immunoaffinity purify complexes containing α4 subunit. (**B**) After concentration, the purified material was subjected to a fractionation by size in a Superdex200 column. The α4 subunit was detected with anti-Flag antibody. (**C**) Polypeptides present in eluted fractions 20 and 25 were detected by silver staining after concentration and SDS-PAGE. The indicated bands of fraction 25 were analyzed by mass spectrometry. Identified polypeptides are marked on the side. Probable contaminants: *** Nut1, negative regulator of Urs2 of the HO promoter, ** Rga1 (RhoGAP for Cdc42); * Tdh, glyceraldehyde-3-phosphate dehydrogenase.

We were interested in learning more about the composition of the detected small intermediates containing α subunits. Since apparently the complexes containing α4 were the most abundant, we focused on these intermediates. Using a strain expressing a variant of this protein carrying the tandem affinity FLAG-6xHis (FH) tag, we performed an affinity purification followed by a gel filtration (Figure 2A,B). The purified proteins that eluted in fraction 20, largely corresponding to 20S CPs, and in fraction 25, corresponding to a smaller complex, were concentrated and subjected to SDS-PAGE and silver staining. 

Bands derived from fraction 25 were excised from the gel and analyzed by mass spectrometry (Figure 2C). The main band corresponded to the α4 subunit. Three bands corresponded to proteins without apparent link to the proteasome and, thus, are likely contaminants (see legend to Figure 2C). Two additional bands yielded peptides from Ssa1, Ssa2, and Sse2: heat shock proteins belonging to the Hsp70 and Hsp110 families, respectively. Hsp70 proteins are a class of cytosolic chaperones involved in many cellular processes, because they assist in the proper folding of newly synthesized proteins (reviewed in [36,37,38]). Ssa1 and Ssa2 are members of an essential subclass of Hsp70 comprising Ssa1, Ssa2, and Ssa3, and Ssa4. Sse1, and Sse2 are paralogs belonging to the Hsp110 chaperones [39]. They act as nucleotide exchange factors (NEF) for Hsp70 proteins [40,41,42] and have an important role in preventing misfolding and aggregation of their substrates by keeping them in a folding-competent state acting as ‘holdases’ [43,44]. 

Proteasome PCs associated with the Hsp70 chaperones were first observed for mammalian proteasomes [45]. More recently, CP assembly intermediates associated with Ssa1 and Ssa2 were also detected in *S. cerevisiae* [46]. These chaperones were detected in high MW complexes containing α4 [47]. The authors proposed that these structures were likely rings of α4 subunits. Additional data suggest that Ssa1/2 are involved in the formation/stabilization of a complex called sub-13S species, a complex containing a subset of α and β subunits [46]. 

### 3.3. Effect of SSE1 Deletion on Small Complexes Containing α4 Separated by Native PAGE 

**Figure 3:**
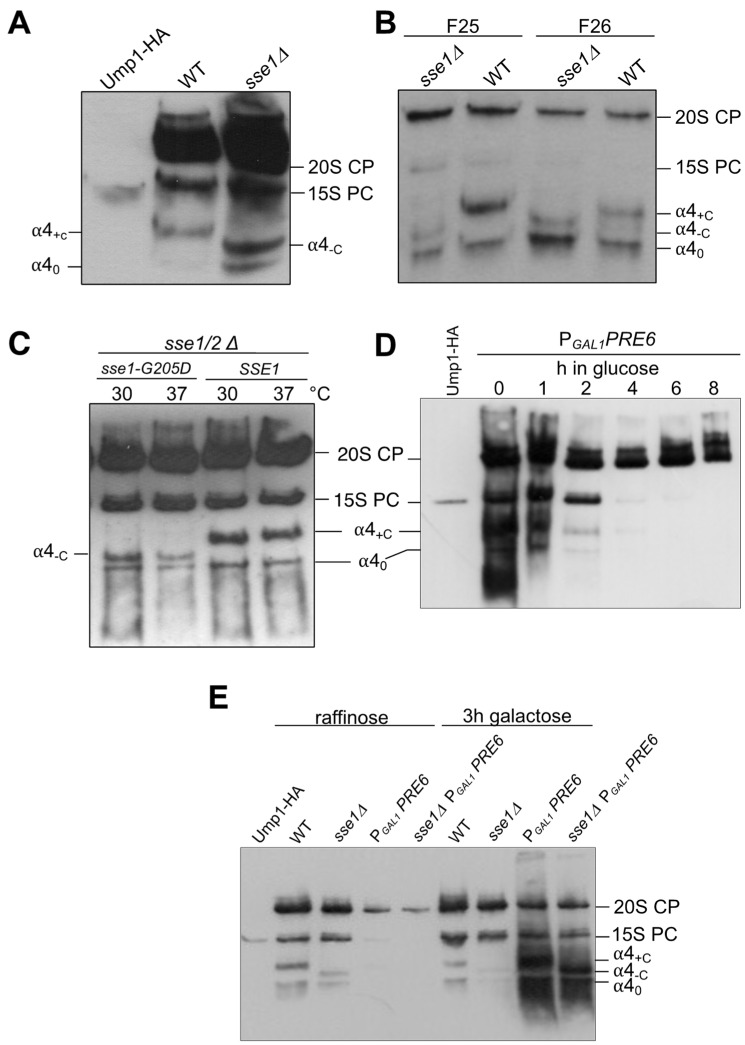
Identification of three distinct small complexes containing α4-HA by native PAGE and anti-HA immunoblotting. (**A**) Total protein extracts (8 μg) from WT and *sse1∆* strains expressing *PRE6-HA* (encoding α4-HA) were compared by native PAGE analysis. (**B**) Fractions 25 and 26 from Superdex200 gel filtration of WT or *sse1∆* protein extracts were analyzed by native PAGE. (**C**) α4 complexes were compared in *sse1∆ sse2∆* cells expressing either WT *SSE1* or *sse1-G205A*, the latter encoding a mutant Sse1 protein with reduced ability to interact with Ssa1/2. The cells were grown at the indicated temperatures for 3 h. (**D**) Cells from a strain expressing *PRE6-HA* from P*_GAL1_* were grown in media containing galactose until OD_600_ of 0.6, washed and grown further in media containing glucose for the indicated times. (**E**) WT or *sse1∆* cells expressing *PRE6-HA* from P*_GAL1_* were grown in media containing raffinose until OD_600_ of 0.6, and switched for 3 h to galactose media. α4_0_, basal complex containing α4; α4_-C_, complex containing α4 lacking chaperone; α4_+C_, complex containing α4 with chaperone. Strains used in this figure are derivatives of BY4741.

In order to confirm the involvement of the Hsp70 and Hsp110 family chaperones in the formation of complexes containing α4, we followed this subunit in mutants impaired in the function of these chaperones. Deletion of the *SSE1* gene causes slow growth, whereas *sse2* null mutants have apparently no growth defect. The combination of both deletions is lethal [48]. We expressed HA-tagged α4 in an *sse1∆* strain, and compared the total protein crude extracts from this strain and the respective WT by native PAGE. As expected, HA signals were detected in the 15S PC, as well as in the 20S and 26S proteasomes, in both backgrounds. In addition, in the WT, α4 was detected in two fast migrating bands, named *α4 complex 0* (α4_0_) and *α4 complex +C* (α4_+C_) (Figure 3A). Strikingly, in the *sse1∆* mutant, while the band corresponding to α4_0_ was detected, the band α4_+C_ was absent. Instead, a band with an intermediate migration was detected and designated *α4 complex -C* (α4_-C_) (Figure 3A). Extracts from the WT and *sse1∆* cells were fractionated by Superdex200 gel filtration. Fractions 25 and 26 were further analyzed by native PAGE and immunoblotting, which confirmed the occurrence of the three earlier complexes containing α4 (complex 0, -C, and +C) (Figure 3B). In another experiment, we used a strain, in which the double deletion *sse1∆ sse2∆* was rescued by expression of either WT *SSE1* or a mutant form of it encoding *Sse1-G205D* with reduced ability to interact with Ssa1/2p [40]. In the sse1-G205D mutant, we again observed the absence of the α4_+C_ band and instead the presence of α4_-C_ (Figure 3C). 

To prove that the detected complexes were real intermediates and not possibly breakdown products of 15S PC or 20S CP occurring during the analysis by native PAGE, we prepared a strain, in which the promoter of the α4-encoding *PRE6* gene was switched to P*_GAL1_*, and the α4 subunit tagged with HA. Cells grown in galactose overexpress *PRE6* and grow like WT. A switch to glucose results in the repression of α4 subunit synthesis. We followed the repression process over time and could observe that 20S CPs, once formed, did not disappear over a longer period of time. By contrast, similar to the short-lived 15S PC, the newly identified α4-containing complexes rapidly disappeared and were hardly detectable after 2 h of repression (Figure 3D). On the other hand, when either WT or *sse1∆* cells expressing *PRE6*/α4 from P*_GAL1_* were pre-grown in raffinose and then shifted to galactose, massive amounts of complexes α4_0_ and α4_+C_ or α4_0_ and α4_-C_, respectively, accumulated (Figure 3E). Overexpression of α4 likely resulted in the accumulation of these complexes because of a lack of corresponding amounts of the next neighboring subunits that would allow the biogenesis to procced.

### 3.4. Effects of sse1 and ssa1-4 Mutations on Complexes Containing Other α Subunits

**Figure 4:**
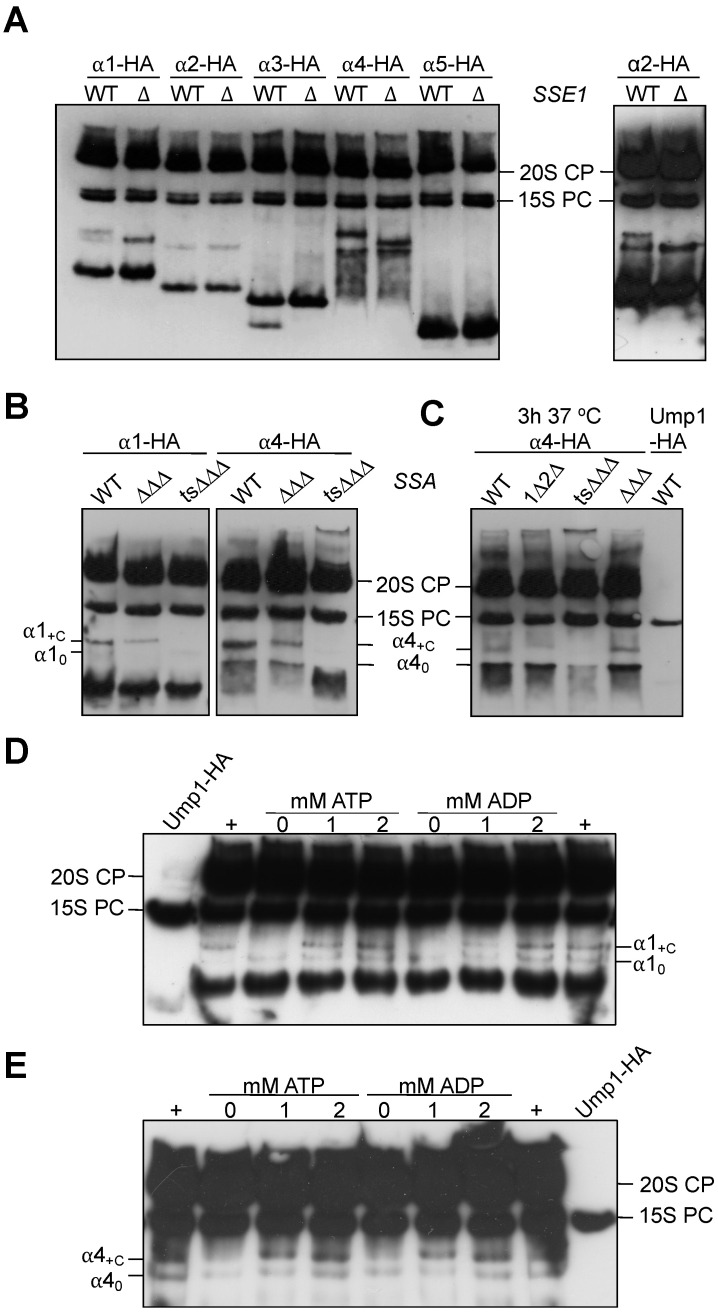
Effects of Hsp70 or Hsp110 mutations and ATP or ADP on early precursors complexes. (**A**) Comparison of the mobility of complexes containing a C-terminally HA-tagged version of the indicated proteasome α subunit in WT and *sse1∆* by native PAGE. 8 μg of total protein were loaded per lane. Right panel, analysis of 16 μg of total protein of the indicated strains expressing α2-HA. (**B**,**C**) Influence of Hsp70 mutations on α1 and α4 complexes analyzed by native PAGE using the following mutants: *ssa1∆ ssa2∆* (∆1∆2); *ssa2∆ ssa3∆ ssa4∆* (∆∆∆); *ssa1-ts ssa2∆ ssa3∆ ssa4∆* (ts∆∆∆). In (**C**), the cells were incubated for 3 h at 37 °C. (**D**,**E**) Cells expressing either α1-HA (**D**) or α4-HA (**E**) were subjected to total protein extraction with 26S buffer without ATP (0 mM). Freshly prepared solutions of ATP or ADP were added to aliquots of the extracts to a final concentration of 1 mM and 2 mM as indicated followed by an incubation at room temperature for 30 min. The samples were analyzed by native PAGE and anti-HA immunoblotting. For comparison, extracts of the same strains were prepared in complete 26S buffer containing 2 mM ATP and loaded at the sides (+).

As described in Section 3.1, aside from α4, we also detected α1, α2, and α5 in low molecular weight complexes in our gel filtration analysis (Figure 1A). We therefore decided to extend our analysis of the effects in the *sse1∆* background to these α subunits. To this end, we compared complexes involving these subunits formed in the WT and *sse1∆* by native PAGE analysis. In addition to the α4 complexes described in Section 3.2 and Section 3.3, this analysis confirmed the presence of precursor complexes containing α1 and α2 subunits in the WT (Figure 4). We found that the absence of *SSE1* also influenced the slower migrating complexes containing α1 or α2 (Figure 4A). Due to the low abundance, α2 patterns were difficult to follow, but when higher amounts of protein were loaded, we could also confirm the absence of the slow migrating band in the *sse1∆* background (Figure 4A, right panel). Taken together, the results establish an interaction and complex formation of Hsp110 chaperones with CP subunits α1/Scl1, α2/Pre8, and α4/Pre6.

Prompted by our identification of Ssa chaperones in our mass spectrometric analysis of α4 complexes (Figure 2C), we next investigated whether mutations impairing functions of Ssa family Hsp70 chaperones would also result in detectable effects on the formation of early complexes with α subunits. As complexes containing α2 were difficult to follow, we studied the influence of the Ssa subfamily members only for complexes involving α1 and α4 (Figure 4B). The Ssa subfamily of Hsp70s is constituted by four non-essential genes, which however cannot be deleted simultaneously, indicating that the four encoded proteins exert a redundant and essential function. *S. cerevisiae* Ssa1 and Ssa2 are constitutively expressed Hsp70s, whereas Ssa3 and Ssa4 are stress-induced [49]. To study the involvement of Ssa family chaperones in formation of the observed complexes, we used strains carrying a triple deletion *ssa2∆ ssa3∆ ssa4∆* (∆∆∆) combined either with WT *SSA1* or with a temperature-sensitive (ts) version of *SSA1* (*ssa1-ts ssa2∆ ssa3∆ ssa4∆*, alias ts∆∆∆). We HA-tagged α1 and α4 in these strains and the respective WT. Protein crude extracts were analyzed by native PAGE and anti-Ha Western blotting. Compared to the WT, the absence of Ssa2, Ssa3, and Ssa4 (∆∆∆) resulted in a reduction of +C complexes containing α1 or α4 (Figure 4B). Even more strikingly, detection of complexes α1_+C_ and α4_+C_ was completely abolished in the ts∆∆∆ mutant. Further experiments with the same mutants, wherein the cells were shifted to 37 °C, confirmed these observations (Figure 4C). Together these results implicate the Ssa chaperone subfamily in the formation of certain complexes with α1 and α4 proteasome subunits. 

In vitro, ATP is required for the formation of a stable complex between Sse1 and Ssa1, while ADP is less efficient [50]. The nucleotides not only induce an Sse1 conformation that allows binding to Ssa1, but are providing direct bridging interactions between the nucleotide binding domains of each HSP [51]. Therefore, if Sse1 and Ssa1 indeed engage in the formation of certain complexes with α1 and α4 subunits, these complexes should respond to the presence or absence of the nucleotides ATP or ADP. Our protein extracts were typically prepared in a buffer containing 2 mM ATP (see Section 2.2). We asked whether the lack of ATP in the extraction buffer would abolish any of the observed complexes, and whether adding ADP or ATP would promote the formation of complexes. Indeed, only the basal bands α1_0_ or α4_0_ were detected in protein extracts prepared in absence of ATP (Figure 4D and Figure 4E, respectively). The addition of either 1 mM or 2 mM of ATP or ADP to the same extracts, however, restored the presence of the complexes α1_+C_ and α4_+C_ (Figure 4D,E). Together, these results indicated that subunits α1 and α4 are present in complexes, formation of which depends on Sse1, Ssa1, and nucleotides. The band pattern analysis suggests that the forms designated as +C contain both chaperones Ssa1 and Sse1, and the form designated as α4_-C_ lacks Sse1 but contains Ssa1. Interestingly, in the native PAGE, the α1 subunit, besides the form α1_0_, was detected in high amounts as a free form. The α4 subunit, in contrast, does not appear to exist long enough as a defined free form to be detectable by native PAGE analysis of extracts, which is in accordance with the results from gel filtration analyses (Figure 1A). In native gels, this subunit occurs in smears, and the first clearly detectable forms are the α4_0_ or α4_C_ complexes. Curiously, when expressed in *E. coli*, α4 is mainly insoluble (Figure S3). The results suggest that Hsp70 and Hsp110 chaperones cooperate in a nucleotide-dependent manner in promoting the folding of α1 and α4 subunits or in keeping them in an assembly-competent state. 

### 3.5. Formation of α1 Complexes Is Independent of α4 and Vice Versa 

**Figure 5:**
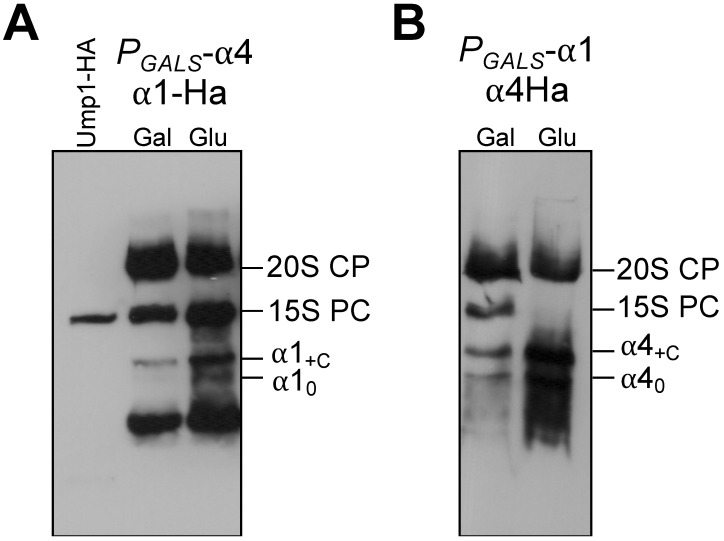
α1_+C_ and α4_+C_ complexes form independently of each other. (**A**) Cells expressing PRE6 (encoding α4) from P*_GALS_* promoter with α1-Ha tagged were grown in galactose (Gal) media and switched to glucose (Glu)-containing media for 15 h. Total protein extracts were analyzed by native PAGE and anti-HA immunoblotting. (**B**) same as in (**A**), but with a strain expressing α1 from P*_GALS_*, and with α4 Ha-tagged.

In the mature proteasome, α1 and α4 subunits are not direct neighbors. Nevertheless, because the mobilities of the α1 and α4 containing complexes in native gels were similar, we asked whether the observed complexes contained both subunits simultaneously. To clarify this, we prepared a set of strains expressing the genes encoding these subunits from the *GALS* promotor. In galactose media, the cells will behave like WT. Long periods of incubation in media containing glucose will shut down the expression of the gene resulting in a depletion of the respective subunit. Protein extracts from these strains were analyzed by native PAGE and immunoblot. Depletion of α4 did not affect the formation of α1-containing complexes. Both forms, α1_0_ and α1_C_, were detected. Reciprocally, depletion of the α1 subunit also did not affect the formation of complexes containing α4 (Figure 5). The latter result was consistent with the absence of α1 in the mass spectrometric analysis of the α4-containing complex (Figure 2C). We conclude that complexes formed from Ssa-Sse chaperones and either α1 or α4 form independently from each other, indicating that these chaperones separately promote the assembly of distinct α subunits. 

The above-mentioned results excluded the idea of the existence of a heteromeric early complex containing both α1 and α4. Interestingly, however, although the complexes apparently form independently, each of them accumulated in the absence of the depleted other subunit. A likely explanation is that such complexes accumulate if downstream steps in the biogenesis involving the respective other subunits are impaired. 

### 3.6. Genetic Interactions between sse1∆ and Mutations Affecting Proteasome Assembly

**Figure 6:**
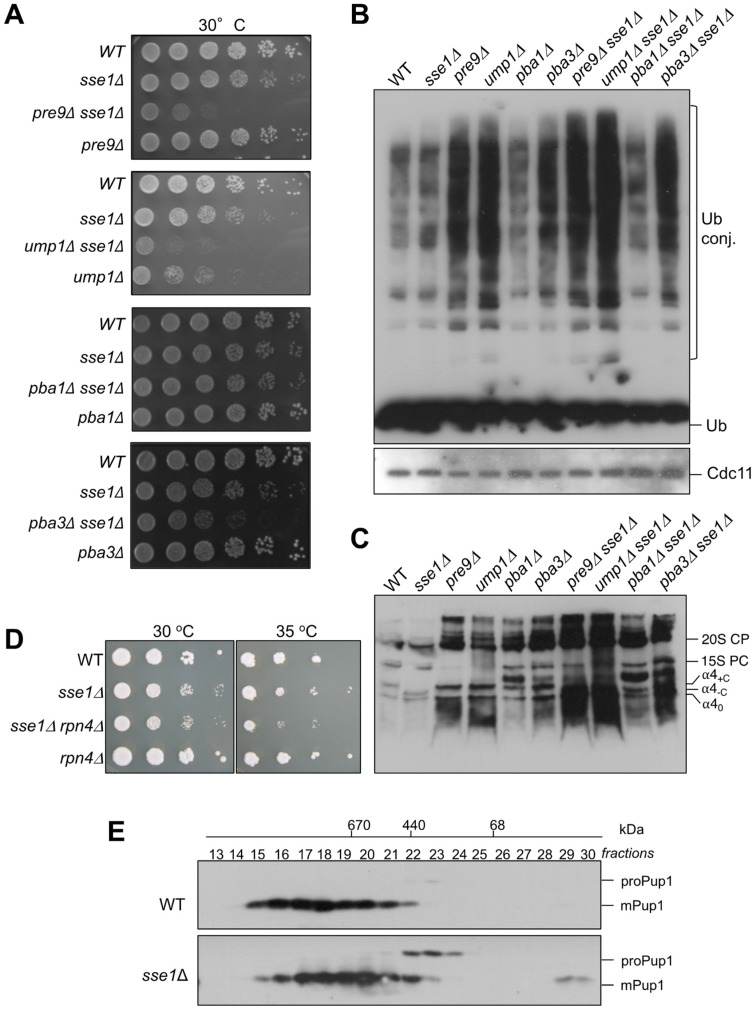
Phenotypes caused by proteasome-related mutations in combination with *sse1∆*. (**A**) Genetic interactions of *sse1∆* with proteasome mutations. (**B**) Evaluation of the amounts of ubiquitylated conjugates in the indicated strains. Extracts were analyzed by SDS-PAGE and anti-ubiquitin Western blotting. Cdc11 was detected to control for comparable protein loading. Positions of free ubiquitin (Ub) and Ub-protein conjugates (Ub-P) are indicated. (**C**) Comparison of complexes containing α4-HA in single and double mutants by native PAGE. (**D**) Growth phenotypes of *rpn4∆* and *sse1∆* single and double mutants. (**E**) Proteasomal complexes containing HA-tagged subunit β2/Pup1 or its precursor form (proPup1) were analyzed by Superose6 gel filtration and anti-HA Western blotting, as described previously [16]. The numbers of fractions (13 to 30) used in the analysis and the positions of molecular weight standards (kDa) used to calibrate the column are indicated at the top of the panel.

The α4 subunit is known to replace the non-essential α3/Pre9 subunit in its absence [13]. Here we showed that a mutation in the *SSE1* gene cause a change in the mobility of complexes containing the α4 subunit. We therefore wanted to test whether the combination of both mutations would lead to an effect on α4 complexes. First of all, we observed a striking synthetic growth defect in the *sse1∆ pre9∆* double mutants that was similarly severe as the one of *sse1∆ ump1∆* (Figure 6A). This growth phenotype goes along with a strong accumulation of ubiquitin conjugates in *sse1∆ pre9∆* cells in comparison to the single mutants or the WT (Figure 6B). Curiously, when we analyzed the complexes containing α4 subunit in the *pre9∆* mutant by native gels, we found that α4_0_ and α4_-C_ accumulated massively in comparison to the WT (Figure 6C). We conclude that the formation of small α4 complexes is independent, not only from α1 (see Figure 5B), but also from α3. 

*PRE9* deletion combined with a deletion of *RPN4* causes lethality [18], indicating that the Rpn4 response is activated in *pre9∆* cells. As a consequence, α4 amounts are elevated resulting in higher amounts of α4 intermediates. In line with this notion, a combination of *RPN4* deletion with *SSE1* deletion would be expected to exhibit also a synthetic effect. Indeed, the double mutant *sse1∆ rpn4∆* grows slower than the single mutants, especially at elevated temperatures (Figure 6D). 

In a similar approach, we asked if there would be a synthetic effect between the deletion of *SSE1* and the absence of proteasome-specific chaperones Ump1, Pba1-Pba2, or Pba3-Pba4. With the exception of *pba1∆ sse1∆* mutant, where the effects were mild, the double mutants, *ump1∆ sse1∆* and *pba3∆ sse1∆,* showed strong synthetic growth defects. In line with these growth phenotypes, crude extracts from the same cells analyzed by native PAGE exhibited higher amounts of small complexes containing the α4 subunit (Figure 6C). These phenotypes were also corroborated by an accumulation of higher amounts of ubiquitin conjugates in the double mutants than in the single mutants (Figure 6B). 

Together our data indicate that complexes α4_0_ and α4_+C_ are formed very early in the assembly pathway, before the action of the chaperones Ump1, Pba1-Pba2, and Pba3-Pba4, or the incorporation of the α3 subunit. The fact that the cells in the tested double mutants with *sse1∆* grew poorly and accumulated ubiquitylated proteins could at least in part be due to the observed roles of Sse1 and Ssa1 for correct folding and efficient assembly of distinct α subunit (α1, α2, and α4). We additionally asked whether a more direct effect of the deletion *SSE1* on proteasome biogenesis can be detected. To address this question, we compared proteasomal complexes containing an HA-tagged version of the β2/Pup1 subunit in WT and *sse1∆* cells by gel filtration (Figure 6E). This subunit is synthesized in a precursor form with an N-terminal pro-peptide (proPup1). Autocatalytic maturation of proPup1 by removal of the pro-peptide occurs when two 15S PC dimerize to form the 20S CP [16]. Compared to the WT strain, *sse1∆* cells accumulated strikingly increased amounts of proteasomal precursor complexes containing the unprocessed β2 subunit (Figure 6E). These results underscore that Sse/Hsp110 chaperones are required for normal efficiency of proteasome assembly.

## 4. Discussion

In the final well-defined and organized barrel-shaped 20S CP structure composed of 28 subunits, each single subunit interacts permanently with several others. For example, the yeast α4 subunit has the largest contact interface with α3 (buried area: 1592 Å), but it also interacts through a large surface with α5 (buried area: 1309 Å), and with the β4 and β5 subunits, although with the two last ones only through much smaller surfaces (buried area: 396.8 Å and 299.7 Å, respectively) [52]. Curiously, in the absence of α3, two α4 subunits can associate with each other enabling substitution of the α3 subunit in the mature CP [13]. This capacity of the α4 subunit probably reflects a self-interacting property of evolutionary predecessors. It is predictable then for each single subunit, that several initial interactions could be possible. The structure of each subunit must contain precise information to which of the many possible neighbors it should bind first, a choice likely driven by the fact that strong interactions give intermediates with the highest stability. Whereas in *Thermoplasma*, the α subunits have the property to homo-oligomerize spontaneously. In a slightly more complex subunit system as the one from *Rhodococcus*, the two α subunits do not directly associate, but can interact productively with both β subunits. Only then, the α-β heterodimers assembly to produce functional proteasomes [24]. In comparison to *Thermoplasma*, the subunit complexity is increased 7-fold in *S. cerevisiae*. This higher complexity goes along with an involvement of the dedicated chaperones Ump1, Pba1-Pba2, and Pba3-Pba4. Although the general 3D structure of each of the seven eukaryotic α subunits is very similar, they might have preserved both the capacity to self-assemble spontaneously and to productively interact with their final neighbors. During evolution, the cells must have chosen the most efficient route for the assembly, along which the final partners from each α and β subunits are selected and correctly combined in a 15S PC. Dedicated chaperones as well as the general chaperones identified in the present work help to achieve or keep conformations of subunits that are competent for interaction and assembly with other subunits. 

### 4.1. α Subunits Initiate Assembly of Proteasome Core Particles without Forming a Heptameric α Ring Intermediate

The formation of an α ring as a template for β subunit incorporation is the currently prevailing model for eukaryotic proteasome assembly [9,10,11]. In our systematic analysis by gel filtration size fractionation of proteasomal complexes from strains, in which one of the 14 α or β subunits was epitope-tagged, however, we did not detect such an α ring intermediate in yeast cells (Figure 1 and Figure S2). Still, the existence of a hetero-heptameric α ring intermediate in the assembly pathway cannot entirely be excluded because it might be just too short-lived to be detectable in our analyses. Our study, however, revealed that α1, α2, and α4 subunits were present in small complexes with molecular weights below 230 kDa. Further analysis of these complexes by native PAGE suggested that each of these subunits was present in complexes with distinct mobilities (Figure 4A). Interestingly, α1 and α2 additionally exist abundantly as free forms, which is in contrast to α4 that was hardly detectable as a free subunit. The β subunits did not reveal any intermediates containing these subunits within the small intermediates range. The finding that some α subunits are found in precursors with lower molecular weight than the β subunits is consistent with the idea that the former initiate proteasome assembly, even though formation of an α ring intermediate is not detectable in wild-type yeast cells. 

### 4.2. The Role of Hsp70/Hsp110 Machinery in Early Proteasome Assembly Steps

Our study reveals that Hsp70 and Hsp110 chaperones contribute to efficient assembly of early complexes involving α1, α2, and α4 subunits. These chaperones likely promote proper folding of the nascent subunits and/or prevent spontaneous self-association or aggregation. The ability to self-assemble is evident from in vivo data showing that two α4 subunit can incorporate next to each other in an α ring in strains lacking the α3 subunit [13,47]. The chaperones Hsp70 and Hsp110 might prevent surfaces of α4 from engaging in such self-interactions to promote proper assembly. In the hetero-heptameric ring of α subunits of the eukaryotic CP, each interface between two neighboring subunits, despite overall similarities, is different. The surface of α4 interacting with α3, for example, bears a loop without defined secondary structure elements (residues 47–62) [12], which is different from the corresponding surfaces of other α subunits. Such subunit-specific features maybe transiently protected by interactions with the chaperones until they find their proper interaction surface. Improper interactions are likely to be more unstable than correct ones initially, allowing chaperones to engage again after dissociation. This way, the chaperone may increase the efficiency of correct interactions within the assembly pathway [53]. Work with human cells indicated that formation of alternative CP forms with two α4 subunits per α ring may occur as a consequence of changes in cell physiology, e.g., caused by cancer mutations, and lead to an increased resistance to stress caused by toxic metals [54]. Based on these results, it is tempting to speculate that a reduced availability of Hsp70 and Hsp110 chaperones, as it can occur under conditions of proteotoxic stress, may also be a factor that influences the ability of α4 subunits to self-assemble and thus to engage in the formation of alternative proteasomes.

Aside from the above-mentioned observation made for α4 in yeast and human cells, expression of protozoan *Trypanosoma brucei* α5 [55] or human α7 [56] in *E. coli* were reported to result in the formation of two or four stacked homo-heptameric rings, respectively. These findings demonstrated that multiple surfaces also of these subunits promote homomeric interactions either within a ring or between rings. The yeast α1, α2, and α4 subunits may similarly also be able to self-associate forming unproductive homomers. The observation that complexes containing individual ones of these subunits, but lacking the Hsp70 and Hsp110 chaperones (designated “αx_0_”), eluted from a gel filtration column between the 67 kDa (albumin) and the 232 kDa markers (catalase) is consistent with this possibility. Our data are insufficient to estimate the oligomeric states of the subunits in these complexes. Whether they correspond to homo-heptameric rings (~200 kDa) as reported for other α subunits (see above) has to be further investigated. Another open question is whether the observed αx_0_ complexes are dead-end products or could serve as a resource that may release free subunits for proper CP assembly under certain conditions, possibly assisted by Hsp70 and Hsp110 chaperones [57].

We have shown here that α1 and α4 form complexes with Ssa1 and Sse1 in an ATP/ADP manner. Hsp70-assisted polypeptide folding is thought to occur in ADP/ATP-dependent cycles with substrate binding occurring in the ADP-bound state, whereas substrate release occurs when the chaperone is in the ATP-bound state [50,51]. For Sse1, it has been suggested that the main functional state is the ATP-bound one [51]. Some studies suggest that Hsp110s act as “holdases” binding directly to denatured proteins [44], and others indicate that Hsp110 is directing substrates to the Hsp70 chaperone [41]. However, the main function of Hsp110 is apparently the catalysis of nucleotide exchange on Hsp70, and it seems that Hsp110 must be bound to ATP to associate with Hsp70 [50]. In our analysis, distinct complexes bearing subunits α1, α2, or α4 subunits were detected that depended on the presence of Hsp70 and Hsp110 (indicated “+C” in Figure 3 and Figure 4). Gel filtration analysis of these α4-containing complexes indicated that they eluted between the 67 kDa and the 232 kDa size markers (Figure 1A and Figure 3B). The calculated molecular mass of a complex of α4 (28.4 kDa), Ssa1 (70 kDa), and Sse1 (77.3 kDa) is 175.7 kDa, which would be consistent with the observed fractionation behavior. For the α4 subunit, an additional complex was observed in the absence of Sse1 (indicated “-C” in Figure 3 and Figure 4), which was not detected in a strain impaired in the function of the Ssa1 family of Hsp70 chaperones. This observation suggested that α4, in contrast to α1, can bind Hsp70 without Hsp110. In line with these interpretations, in presence of the Sse1-G205D mutant, which has low affinity to Ssa1, the complex α4+_C_ disappeared giving rise to complex α4_-C_ (Figure 3C). 

Taken together, our identification of complexes of α1, α2, or α4 subunits with Hsp70 and Hsp110 suggests that these two chaperones might cooperate to prevent unproductive homomeric interactions of these α subunits and thereby increase the efficiency of CP assembly. Consistent with this notion is the observation that increased amounts of precursor complexes were detected in cell lacking Sse1 (Figure 6E). Dissection of the molecular details of Hsp70-Hsp110-assisted proteasome assembly may help to understand the causes and consequences of defective proteasome biogenesis in various diseases [1,2,4,5,6,7].

## Data Availability

Data is contained within the article or Supplementary Materials.

## Supplementary Materials

The following supporting information can be downloaded at: https://www.mdpi.com/article/10.3390/biom13010011/s1, Figure S1: Gel filtration analysis of α4 and α6; Figure S2: Gel filtration analysis of proteasome β subunits; Figure S3: The subunit α4 synthesized in *E. coli* is largely insoluble; Table S1: Strains used in this work. Refs. [8,21,40,41] are cited in Table S1.
