# Supplementary material for: Hsp70 and Hsp110 Chaperones Promote Early Steps of Proteasome Assembly
Source: Biomolecules. 2022 Dec 21;13(1):11. doi: 10.3390/biom13010011 (PMC9855889; doi:10.3390/biom13010011)
Supplement: Supplementary file 1 [file biomolecules-13-00011-s001.zip › biomolecules-2097602-supplementary.pdf]

Table S1 - Strains used in this work

strain name	genotype	Derivative of	reference
AM136	*MAT*a *PRE2* (β5)-*HA*::*YIplac211*	JD47-13C	this work
AM200	*MAT*a *ump1Δ::HIS3MX6 sse1Δ::KanMX6*	JD47-13C	this work
AM198-1D	*MAT*a *pba3Δ::kan sse1Δ::Kan*	JD47-13C	this work
BY4741	*MAT*a *his3-Δ1 leu2-Δ0, met15-Δ0 ura3--Δ0*	"WT"	Euroscarf
CM2	*MAT*a *PRE6* (α4)-*HA*::*YIplac211*	JD47-13C	this work
CM3	*MAT*a *PRE6* (α4)-*HA*::*YIplac211 ssa1-ts45 ssa2Δ::LEU2 ssa3Δ::TRP1 ssa4Δ::LYS2*	JBY68	this work
CM5	*MAT*a *PRE6* (α4)-*HA*::*YIplac211 sse1Δ::KanMX4*	BY4741	this work
CM13	*MAT*a *PRE6* (α4)-*HA*::*YIplac211*	BY4741	this work
CM14	*MAT*a *PRE6* (α4)-*HA*::*YIplac211*	JN54	this work
CM24	*MATα* *PRE6* (α4)-*HA*::*YIplac211 sse1/2Δp414TEFsse1-G205D*	W303	this work
CM25	*MATα* *PRE6* (α4)-*HA*::*YIplac211 sse1/2Δp414TEFSSE1WT*	W303	this work
CM29	*MAT*a *P*$_{GAL1}$*PRE6*-(α4)-*HA*::*YIplac211*	BY4741	this work
CM30	*MAT*a P$_{GAL1}$*PRE6*- α4)-*HA*::*YIplac211 sse1Δ::kanMX4*	BY4741	this work
CM45	*MATα rpn4Δ::HIS3MX6*	ML163	this work
CM46	*MAT*a *rpn4Δ::HIS3MX6 sse1Δ::KanMX4*	BY4741	this work
CM54	*MAT*a *PUP1* (β2)-*HA*::*YIplac211*	BY4741	this work
CM55	*MAT*a *PUP1* (β2)-*HA*::*YIplac211 sse1Δ::KanMX4*	BY4741	this work
CM61	*MAT*a *PUP3* (β3)-*HA*::*YIplac211*	JD47-13C	this work
CM143	*MAT*a *pre9Δ::KanMX4 sse1Δ::HIS3MX6*	Y04765	this work
CM90	*MAT*a *PBA2-HA*::*YIplac211*	JD47-13C	this work
CM128	*MAT*a *PRE4* (β7)-*HA*::*YIplac211*	JD47-13C	this work
CM134	*MAT*a *P*$_{GALS}$*SCL1::kanMX4 PRE6-2HA*::*YIplac211*	JD47-13C	this work
CM152	*MAT*a *PRE7* (β6)-*HA*::*YIplac211*	JD4713C	this work
CM182	*MAT*a *PRE8* (α2)-*HA*::*YIplac211 sse1Δ::TRP1*	PR93	this work
CM185	*MAT*a *SCL1* (α1)-*HA*::*YIplac211 sse1Δ::TRP1*	PR93	this work
CM199	*MAT*a *P*$_{GALS}$*-PRE6* (α4)*::KanMX6 SCL1* (α1)-*HA*::*YIplac211*	JD47-13C	this work
CM252	*MATα SCL1* (α1)-*HA*::*YIplac211 ssa2Δ::LEU2 ssa3Δ::TRP1 ssa4Δ::LYS2*	JN516	this work
CM253	*MAT*a *SCL1* (α1)-*HA*::*YIplac211 ssa1-ts45 ssa2Δ::LEU2 ssa3Δ::TRP1 ssa4Δ::LYS2*	JBY68	this work
CM272	*MATα PRE6* (α4)-*HA*::*YIplac211 ssa1Δ::HIS3 ssa2Δ::LEU2*	MW123	this work
FP6	*MAT*a *PRE3* (β1)-*HA*::*YIplac211*	JD47-13C	Pardelha, FP
FP8	*MAT*a *PRE6* (α4)-*HA*::*YIplac211 pre9Δ::HIS3MX6*	JD305	Pardelha, FP
FP14	*MAT*a *PRE6* (α4)-*HA*::*YIplac211 pre9Δ::HIS3MX6 sse1Δ::TRP1*	JD305	Pardelha, FP
FP16	*MAT*a *PBA4-HA*::*YIplac211*	JD47-13C	Pardelha, FP
JBY68	*MAT*a *ssa1-ts45 ssa2Δ::LEU2 ssa3Δ::TRP1 ssa4Δ::LYS2*	JN54	[41]
JN54	*MAT*a *his3-11,3-15 leu2-3,2-112 lys2 trp1-Δ1 ura3-52*	"WT"	[41]
JN516	*MATα ssa2Δ::LEU2 ssa3Δ::TRP1 ssa4Δ::LYS2*	JN54	[41]
JD47-13C	*MAT*a *his3-Δ200 leu2-3,112 lys2-801 trp1-Δ63 ura3-52*	"WT"	[21]
JD59	*MAT*a *ump1Δ::HIS3MX6*	JD47-13C	[8]
JD127	*MAT*a *PRE1* (β4)-*HA*::*YIplac211 UMP1-HA*::*YIplac128*	JD47-13C	[8]
JD129	*MAT*a *UMP1-HA*::*YIplac128*	JD47-13C	[8]
JD139	*MAT*a *PUP1* (β2)-*HA*::*YIplac211*	JD47-13C	[8]
JD183	*MAT*a *PRE10* (α7)-*HA*::*YIplac211*	JD47-13C	this work
JD305	*MAT*a *pre9Δ::HIS3MX6*	JD47-13C	this work
JM5	*MAT*a *PRE8* (α2)-*HA*::*YIplac128*	JD47-13C	this work
JM6	*MAT*a *PRE9* (α3)-*HA*::*YIplac211*	JD47-13C	this work
JM9	*MAT*a *PUP2* (α5)-*HA*::*YIplac211*	JD47-13C	this work
JM10	*MAT*a *PRE5* (α6)-*HA*::*YIplac211*	JD47-13C	this work
JM12	*MAT*a *PRE6* (α4)-*F6H*::*YIplac204 PRE8* (α2)-*HA*::*YIplac128*	JD47-13C	this work
JM15	MATa PRE6 (a4)-HA::YIPlac211 PRE5 (a6)-HA::LEU2	JD47-13C	this work
JM17	*MAT*a *SCL1* (α1)-*HA*::*YIplac211*	JD47-13C	this work
ML163	*MATα his3-Δ1 leu2-Δ0, lys-Δ0 ura3-Δ0 trp1-Δ0*	BY4742	London, M
MW123	*MATα leu2Δ trp ura3 his3 lys2 ssa1Δ::HIS3 ssa2Δ::LEU2*	JN54	[21]
PG24	*MAT*a *PRE6* (α4)-*HA*::*YIplac211 ump1Δ::HIS3MX6*	JD47-13C	Gouveia, P
PG25	*MAT*a *UMP1-HA*::*YIplac128*	BY4741	Gouveia, P
PR93	*MAT*a *sse1Δ::TRP1*	JD47-13C	this work
PR102	*MAT*a *pba1Δ::HIS3MX6 sse1Δ::TRP1*	JD47-13C	this work
PR108	*MAT*a *PRE6* (α4)-*HA*::*YIplac211 pba1Δ::HIS3MX6 sse1Δ::TRP1*	PR102	this work
PR110	*MAT*a *PRE6* (α4)-*HA*::*YIplac211 pba3Δ::HIS3MX6 sse1Δ::TRP1*	JD47-13C	this work
PR118	*MAT*a *pba1Δ::HIS3MX6*	JD47-13C	this work
PR122	*MATα PRE6* (α4)-*HA*::*YIplac211*	JN516	this work
PR125	*MAT*a *PRE6* (α4)-*HA*::*YIplac211 sse1Δ::TRP1*	JD47-13C	this work
PR126	*MAT*a *PRE9* (α3)-*HA*::*YIplac211 sse1Δ::TRP1*	PR93	this work
PR127	*MAT*a *PUP2* (α5)-*HA*::*YIplac211 sse1Δ::TRP1*	JD47-13C	this work
PR129	*MAT*a *pba1Δ::HIS3MX6 PRE6* (α4)-*HA*::*YIplac211*	JD47-13C	this work
PR132	*MAT*a *PRE6* (α4)-*HA*::*YIplac211 pba3Δ::HIS3MX6*	JD47-13C	this work
PR135	*MAT***a** *PRE6* (α4)-*HA*::*YIplac211 sse1Δ::kanMX6 ump1Δ::HIS3MX6*	JD47-13C	this work
PR170	*MAT*a *SCL1* (α1)-*HA*::*YIplac211*	JN54	this work
W303	*MAT*a *ade2-1 trp can1-100 leu2-3,112 his3-11,-15 ura3*	"WT"	[40]
Y01576	*MAT*a *pba3Δ::KanMX4*	BY4741	Euroscarf
Y02146	*MAT*a *sse1Δ::KanMX4*	BY4741	Euroscarf
Y04765	*MAT*a *pre9Δ::KanMX4*	BY4741	Euroscarf

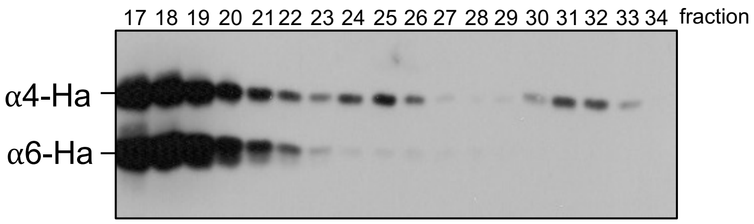

Figure S1. Gel filtration analysis of α4 and α6. A strain was generated expressing both α4 and α6 in an HA-tagged form. Gel filtration (Superdex200) and anti-HA western blot analysis was done as described in Figure 1.

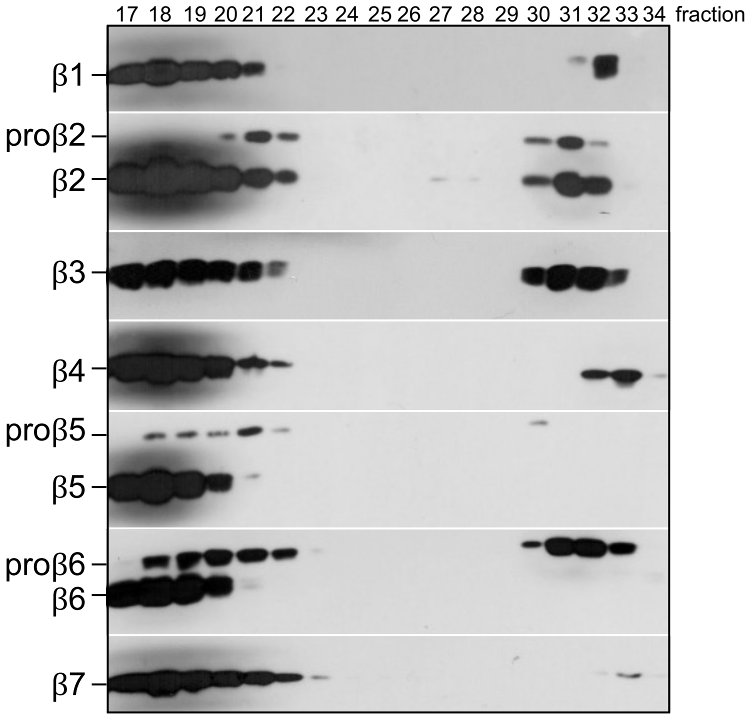

Figure S2. Gel filtration analysis of β subunits. Strains expressing HA-tagged versions of the seven distinct β subunits were used to analyze complexes formed by these subunits by Superdex200 gel filtration and anti-HA western blotting as described in Figure 1. The positions of the mature and unprocessed (pro) form of the β subunits are indicated on the left.

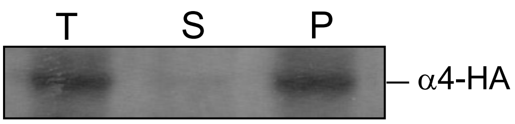

Figure S3. The subunit α4 synthesized in E. coli is largely insoluble. Total protein extract (T) from a bacteria strain overexpressing α4-HA was analyzed by SDS-PAGE. (S) Soluble material. (P) Material found in the pellet.
